# Large Genital Cavernous Hemangioma: A Rare Surgically Correctable Entity

**DOI:** 10.1155/2015/950819

**Published:** 2015-11-29

**Authors:** Goto Gangkak, Anoop Mishra, Shivam Priyadarshi, Vinay Tomar

**Affiliations:** Department of Urology, SMS Medical College & Hospitals, Jaipur, Rajasthan 302004, India

## Abstract

We report a case of 24-year-old male presenting with painless penoscrotal swelling for 3 years. On examination, a large soft bag of worm-like, superficial, nonpulsatile swelling was present in scrotum and penis. Color Doppler showed dilated tortuous vessels and on angiography no connections to corpora or vessels were seen. So a diagnosis of hemangioma was made and a surgical excision was carried out by circumcoronal and scrotal incisions. Postop course was uneventful. At 6 months of follow-up, no recurrence was seen and wound had healed with excellent cosmetic appearance.

## 1. Introduction

Cavernous hemangiomas involving genitalia are a rare clinical entity [1]. Genital hemangiomas have been mostly reported in pediatric age group and much rarely reported in adults [2, 3]. These tumors can involve glans, penile shaft, scrotum, and perineum and can even extend to anterior abdominal and pelvis [4]. Genital cavernous hemangiomas often pose diagnostic and treatment dilemma for the treating surgeon [5]. Various treatment options are available like surgical excision, laser fulguration, intralesional sclerotherapy, and cryotherapy but there is no clear consensus on its management due to its rarity. We report here a case of large cavernous hemangioma involving glans, penile shaft, and scrotum which was successfully treated surgically and review of the literature of various treatment options available.

## 2. Case Presentation

**Figure 1:**
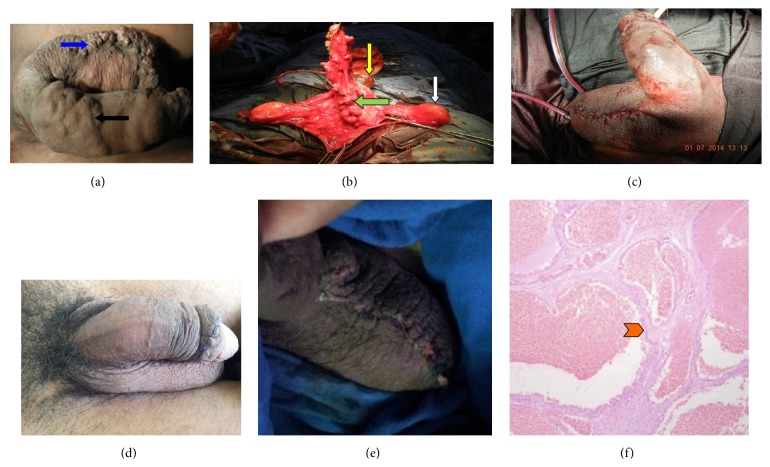
(a) Showing preoperative photograph of the hemangioma: black solid arrow showing the penile component of hemangioma and blue solid arrow showing scrotal extension of hemangioma. (b) Showing intraoperative picture: green solid arrow showing the hemangioma tissue dissected from penile shaft, yellow solid arrow shows glans, and white solid arrow shows the testis. (c) Final operative picture with suction drain in situ. ((d), (e)) Postop picture at follow-up showing healed wound with an excellent cosmetic outcome. (f) Showing microscopic picture (H&E stain) endothelium lined vascular channels with intervening thick fibrous septa (red arrow head shows thick fibrous septa characteristic of cavernous hemangioma).

A 24-year-old male presented with history of progressively increasing penoscrotal swelling for 3 years. It was associated with dull aching pain and discomfort. There was no history of trauma or previous surgery. No significant family history was present. On examination, a bosselated, nontender, nonpulsatile, noncompressible, soft bag of worm-like mass was felt on glans and the penile shaft circumferentially, also extending into the scrotum (Figure 1(a)). No other similar lesions were found elsewhere. The rest of the examination was normal. Blood investigations were within normal limits. A color Doppler revealed multiple dilated tortuous channels limited to skin and separate from corpora. On Doppler blood flow was reported in some of these channels. CT scan revealed that the lesion was limited to penis and scrotum and arteriography images did not reveal any feeding vessels or connections with the arterial vessels. So a surgical excision was planned.

### 2.1. Operative Technique

Patient was given spinal anesthesia and catheterized preoperatively. A circumcoronal circumferential incision which is 1 cm from coronal sulcus was made and dissection was carried deep up to bucks fascia and the hemangiomatous tissue was carefully dissected from buck's fascia and skin of penile shaft (Figure 1(b)). Then through a 6 cm long elliptical incision over the median raphe hemangiomatous tissue was intussuscepted into scrotum. There was no communication seen with corpora and any vessel. Glanular lesion was left untreated. Finally circumcision and primary closure of scrotal skin over a suction drain was done (Figure 1(c)). Postop course was uneventful. On histopathological examination dilated channels lined with endothelium containing red blood cells (RBCs) were seen. The presence of thick fibrous tissue between the vessels was seen, which was characteristic of cavernous hemangioma (Figure 1(f)).

### 2.2. Outcome

At 1-month follow-up no recurrence was seen and wound had healed well (Figures 1(d) and 1(e)). At 6-month follow-up the glanular lesion also had completely resolved with an excellent cosmetic appearance.

## 3. Discussion

Our case of a large cavernous hemangioma of penis and scrotum represents a very rare clinical entity. Many investigators consider them to be congenital vascular anomaly and a benign tumor [6]. Some consider it to be a herniation of cavernosal tissue [7] and others consider it to be due to revascularization from penile hematoma [8].

Since the first report by Boullay in 1851, very few cases have been reported so far [9]. The hemangiomas can be located in glans, penile shaft, and scrotum [8]. Cavernous hemangiomas may be present since birth but mostly they are noted in adolescence as penoscrotal mass or due to concern about the cosmetic appearance as in our case. Usually they are painless but can be associated with pain, ulceration, and bleeding [5]. The lesion usually does not involute with time and can also present with extension into perineum, anterior abdominal wall, and pelvis. Some authors have also shown concern for infertility with these lesions [4]. Rarely they can be associated with hemangiomas at other sites like bladder and rectosigmoid [10] and can be associated with syndromes like Fabry disease and Klippel-Trenaunay syndrome [11].

Imaging studies are useful to identify and delineate the extent of the hemangioma, as well as detection of any associated anomalies. Color Doppler demonstrates blood flow within these lesions but the absence of flow does not rule out the presence of these lesions. Other imaging modalities like computed tomography (CT scan) and magnetic resonance imaging (MRI) are very useful for diagnosis and delineating their relationship with adjacent structures [12].

Treatment decisions have to take into consideration the location of the lesion, size of lesion, cosmetic outcome, and cost of treatment. Among the various therapeutic options, nonsurgical treatments like laser (CO_2_ laser, Nd: YAG laser, and yellow-light laser) and intralesional sclerotherapy (Polidocanol, hypertonic saline) have been used primarily for smaller glanular lesion with satisfactory outcomes [13, 14]. Due to risk of bleeding because of high vascularity and possibility of scar formation, surgical excision of these tumors is not favored [1]. Jimenez-Cruz and Osca [14] first described a successful Nd: YAG laser treatment of a glans penis hemangioma. Ulker and Esen [13] also reported good cosmetic outcome with Nd: YAG laser. However, cost is much higher in laser therapy and carbon dioxide laser is primarily used for small glanular lesions.

Hemal et al. [15] demonstrated successful application of intralesional sclerotherapy with hypertonic saline in glans lesion. Savoca et al. [16] similarly used 2% of Polidocanol with good results. However, caution must be taken when using large volume of sclerosants due to risk of necrosis of the erectile tissues and major complications like thrombophlebitis and pulmonary embolism. Also a clear cut plane between lesion and cavernosal tissue should be demonstrated before embarking on this procedure. Therefore, it is selectively used to treat small lesions on glans penis along with laser therapy sometimes. Goldwyn and Rosoff [17] reported the successful use of cryotherapy for the treatment hemangioma but their use has been limited.

Earlier surgical excision was recommended for all lesions due to risk of traumatic rupture and bleeding but the risk is very low [18, 19]. Cosmesis, persistent symptoms, and risk of infertility justify treatment of these lesions. For large or multiple hemangiomas surgical excision is recommended, because of nonfeasibility of nonsurgical options and possibility of complete removal in one setting. Lesions on the glans are cosmetically important and have poor outcomes by surgical procedures [1], so a conservative treatment and watchful waiting are a viable option. Few authors have reported the surgical excision of these large lesions but the outcome and follow-up reports have been inconsistently reported [1, 6, 20, 21].

In our case, we decided to operate on the patient due to the large size and extent of the hemangioma and an excellent surgical outcome was achieved due to meticulous dissection and hemostasis. Thus surgical excision remains a good option for large and multiple lesions and results are quite satisfactory with good surgical planning and technique.

## 4. Conclusion

Cavernous hemangioma involving penis and scrotum is very rare and it poses both diagnostic and therapeutic challenges for treating physician. Surgical excision is preferable for large and multiple lesions with satisfactory outcomes.
